# Utility of arterial spin labeling for objective assessment of intratumoral microvessels in diffuse hemispheric glioma, H3 G34R-mutant: A case report and literature review

**DOI:** 10.1016/j.radcr.2022.11.074

**Published:** 2022-12-21

**Authors:** Kei Kitakami, Takaaki Beppu, Yuichi Sato, Akira Kurose, Kuniaki Ogasawara

**Affiliations:** a Department of Neurosurgery, Iwate Medical University, 2-1-1 Idai-dori, Yahaba, Shiwa, Iwate Pref., 028-3694, Japan; b Department of Anatomic Pathology, Hirosaki University Graduate School of Medicine, Hirosaki, Aomori Pref., Japan

**Keywords:** Diffuse hemispheric glioma H3 G34-mutant, Arterial spin labeling, Bevacizumab

## Abstract

Imaging findings of diffuse hemispheric glioma H3 G34-mutant (DHG, H3 G34m), a new variant of glioma under the World Health Organization classification, have recently been vigorously debated. Here, we report a case of DHG, H3 G34m in which objective assessments of intratumoral microvessels using arterial spin labeling (ASL) were useful for preoperative diagnosis, selection of anti-tumor drugs, and tracking therapeutic responses. The patient was a 34-year-old woman who presented with weakness in the left arm. Preoperative magnetic resonance imaging (MRI) showed no specific findings of hyperintensity on fluid-attenuated inversion recovery imaging and faint enhancement on T1-weighted imaging with contrast media in the tumor. However, ASL showed a convincing finding of high blood flow in the entire tumor, allowing identification of the tumor as malignant glioma. Tumor specimens obtained from biopsy showed that the tumor comprised low-differentiated tumor cells, abundant histiocytes, and highly dense microvessels. Immunohistochemical findings such as positive findings for H3 G34R and p53, and negative findings for IDH-1, ATRX, and OLIG2 led to the diagnosis of DHG, H3 G34m. Based on findings of hyperperfusion on ASL and detection of vascular endothelial growth factor (VEGF), we administered the anti-VEGF antibody bevacizumab. The tumor shrank significantly but remained. However, the residual tumor showed hypoperfusion on ASL, strongly suggesting tumor remission. Objective assessments of blood flow using ASL are useful in clinical practice for patients with DHG, H3 G34 showing non-specific findings on conventional MRI.

## Introduction

Diffuse hemispheric glioma, H3 G34-mutant (DHG, H3 G34m) has been added as a central nervous system tumor in the World Health Organization (WHO) classification of tumors in 2021, as a new variant of pediatric-type diffuse high-grade glioma of grade 4 [1]. DHG, H3 G34m shows mutation in the H3-3A gene for transcriptional regulation (G34R/V) [1,2]. The histological findings appear mainly either glioblastoma-like with highly cellular proliferation, vascularization and necrosis, or central nervous system embryonal tumor-like with poorly differentiated small cell proliferation [1,3]. However, some DHG, H3 G34m have been reported with other histological findings [4–8]. Immunohistochemically, positivity for H3 G34R leads to a diagnosis of DHG, H3 G34Rm. In most cases, p53 is positive and ATRX is negative. Further, completely negative results for OLIG2 can supplement the diagnosis [1,^2^,^4^,9]. The extent of glial fibrillary acidic protein (GFAP) expression is variable for each case, depending on the degree of tumor cell differentiation. DHG, H3 G34m mainly occurs unilaterally in the cerebrum, particularly in the parietal and frontal lobes in children, but also in adults [4,10]. The median age at diagnosis has been reported as 15 years when subjects were limited to children [11,12], and 19 years for all ages [3,10]. The frequency of DHG, H3 G34m is very low, reportedly representing 3.4% of gliomas in all generations [13]. While DHG, H3 G34m accounts for 16.2% of pediatric hemispheric high-grade gliomas [12], 13.7% of all DHG, H3 G34m cases are diagnosed at more than 30 years old [10]. The frequency of diagnosis is thus lower at mature age than at younger age. Raising DHG, H3 G34m preoperatively as a differential diagnosis is this difficult when the disease occurs in mature patients.

MRI of DHG, H3 G34m demonstrates non-specific findings of signal-hyperintense areas on T2-weighted imaging (T2WI) and fluid-attenuated inversion recovery (FLAIR) imaging. On gadolinium-enhanced T1-weighted imaging (Gd-T1WI), the frequency of tumor enhancement has been reported as 63%-79.2% [8–10]. Patients with DHG, H3 G34m showing non-enhancement are not uncommon, despite DHG, H3 G34m being a grade 4 glioma. On the other hand, calcified foci are observed on imaging in 22.2% of cases [10], but are neither frequent nor specific. The new establishment of this tumor type, low occurrence rate, and non-specific features of conventional neuroimaging may make preoperative diagnosis of DHG, H3 G34m difficult. DHG, H3 G34m is often diagnosed preoperatively as a non-neoplastic disease [4,14]. Supplementary neuroimaging other than conventional MRI is therefore desired for the preoperative diagnosis of DHG, H3 G34m [7]. We encountered an adult patient with DHG, H3 G34m in whom preoperative conventional imaging showed non-specific findings, but arterial spin labeling (ASL) imaging demonstrated hyperperfusion throughout the entire tumor, leading to expansion of the differential diagnosis. ASL also helped us select therapy and keep track of the therapeutic response. While reviewing the preoperative imaging findings, histological findings, and prognosis of DHG, H3 G34m, we discuss the utility of ASL in DHG, H3 G34m showing non-specific findings on MRI.

## Case report

**Fig. 1:**
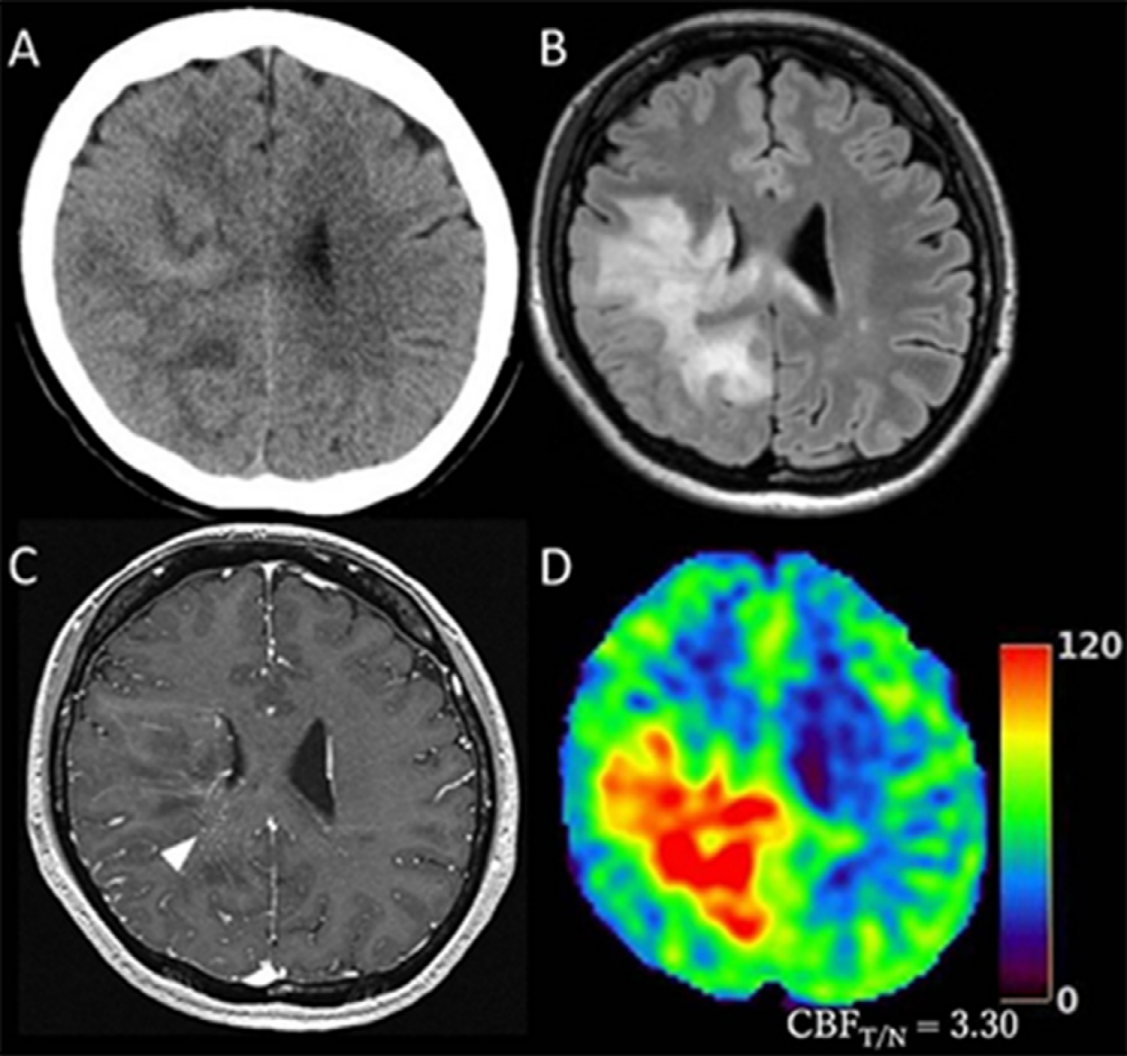
Neuroimaging on admission to our hospital. (A) Non-contrast CT. (B) FLAIR. (C) Gd-T1WI. (D) ASL. Arrowhead represents slight enhancement.

We performed all clinical practices for the present patient in compliance with the precepts established by the Declaration of Helsinki. The patient was a 34-year-old woman with no contributory past or family history. She felt weakness and numbness in the left arm and visited the former hospital. Based on findings from MRI, physicians suspected a high possibility of demyelinating disease and administered one course (3 days) of steroid pulse therapy approximately 3 weeks after the onset of symptoms. However, this proved ineffective and the lesion enlarged along with exacerbation of left hemiparesis and sensory disturbance. The patient was then transferred and admitted to our hospital approximately 2 months after symptoms onset. On admission, the patient had clear consciousness (Glasgow coma scale, 15), but presented with severe hemiparesis of the left extremities at stage 2 of the manual muscle test, and sensory disturbance on the left side of the body. Karnofsky performance status (KPS) was 50%. Non-contrast computed tomography (CT) showed a mild hyperdense area in the center of the lesion, but did not identify any obvious calcification (Fig. 1A). As findings on MRI, FLAIR showed hyperintense areas in the cortex and white matter of the right parietal and frontal lobes, extending to the splenium of the corpus callosum, and Gd-T1WI showed only faint enhancement in a portion of the lesion. In contrast, ASL demonstrated very high blood flow within the entire area corresponding to the hyperintensity on FLAIR imaging (Figs. 1B-D). Mean cerebral blood flow (CBF) was automatically measured for a region of interest 10 mm in diameter placed on the area of highest CBF in the tumor and normal cerebral white matter in the contralateral hemisphere on the ASL map. The ratio of CBF in the tumor to CBF in the normal brain (CBF_T/N_) was 3.30.

**Fig. 2:**
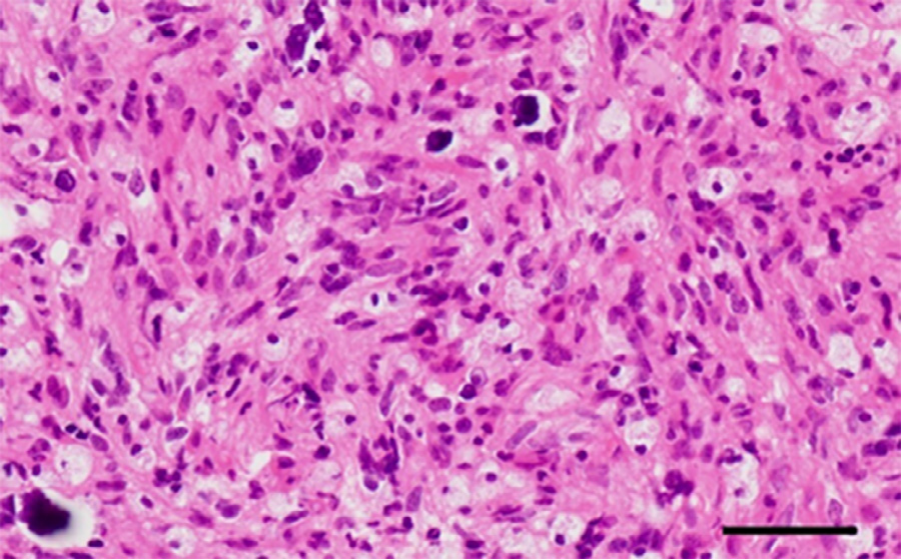
Hematoxylin and eosin (HE) staining. Bar = 50 μm.

**Fig. 3:**
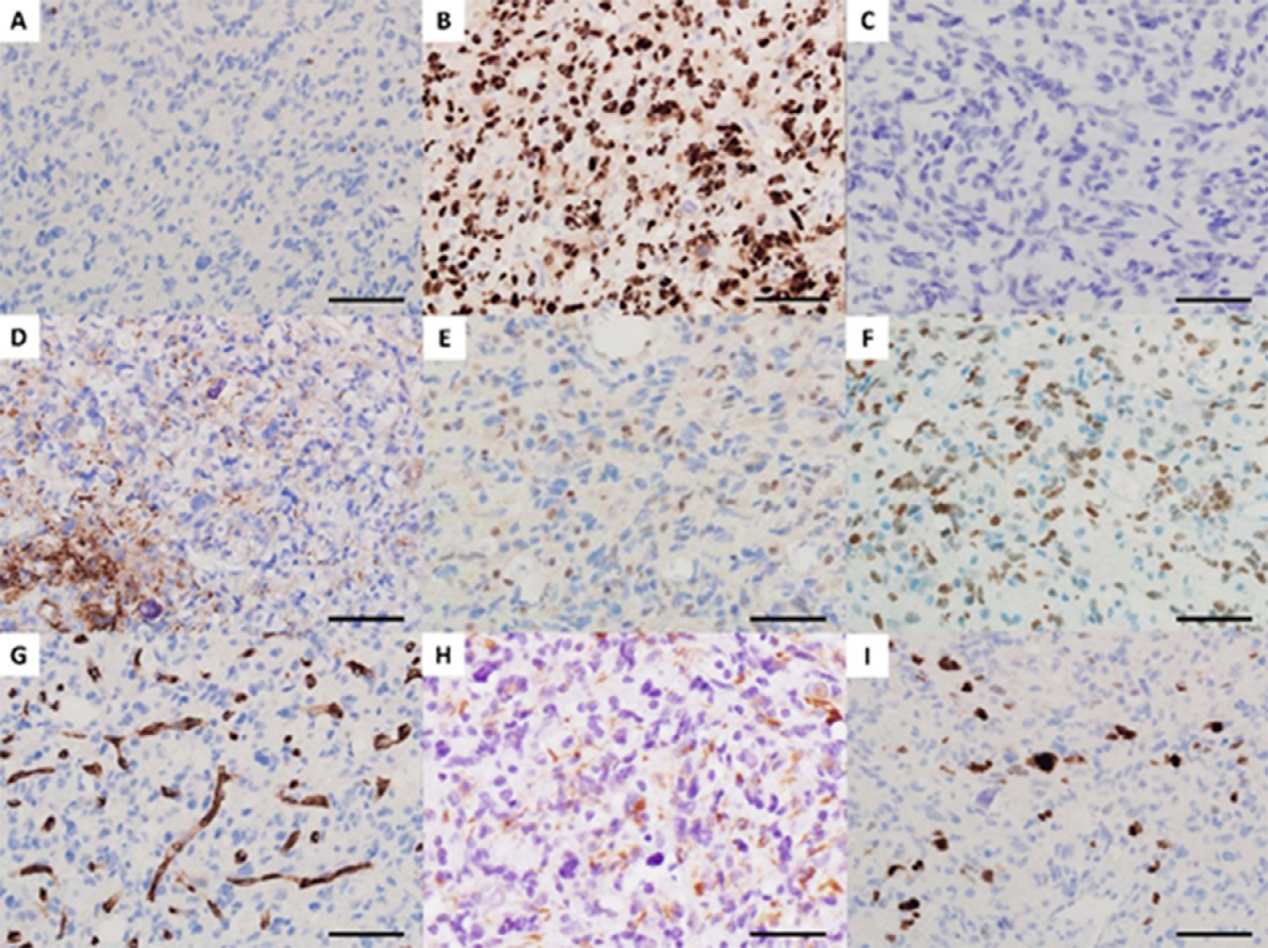
Immunohistochemical results. (A) IDH-1 R132H; (B) p53; (C) OLIG2; (D) GFAP; (E) ATRX; (F) H3 G34R; (G) CD34; (H) VEGF-A; (I) Ki-67. Bar = 50 μm.

Although CT and conventional MRI on admission only showed nonspecific findings, we were able to differentiate the tumor as malignant glioma with vigorous microvascular proliferation in the entire tumor based on the convincing findings from ASL of hyperperfusion within the entire tumor. For pathological diagnosis, we carried out stereotactic needle biopsy under local anesthesia, targeting a faintly enhanced area of the parietal lobe (arrowhead, Fig. 1C). Although specimens obtained from the biopsy were small, pathological findings with hematoxylin and eosin (HE) staining revealed proliferation of low-differentiated tumor cells with relatively small spindle-shaped or irregular oblong nuclei and numerous histiocytes with foamy cytoplasm surrounding the regular round nuclei. In addition, small foci of calcification were sparsely seen within the tumor (Fig. 2). While some small necrotic regions were observed, no tumor-cell palisading was observed around the necrosis. Immunohistochemistry showed negative for IDH-1 and positive for p53, and OLIG2 was completely absent (Figs. 3A-C). GFAP was detected in non-neoplastic fibers, but not in tumor cells (Fig. 3D). ATRX was expressed in the nuclei of vascular endothelial cells and histiocytes, but not in those of tumor cells (Fig. 3E). These findings led us to suspect DHG, H3 G34m. Finally, positive results for H3 G34R led to the diagnosis of DHG, H3 G34Rm (Fig. 3F). Dense networks of CD34-positive chicken-wire-like capillaries without vascular endothelial proliferation were observed within the tumor (Fig. 3G). VEGF expression was detected in tumor cells using anti-VEGF-A antibody (Fig. 3H). Ki-67 was expressed only in the nuclei of tumor cells, with a 16% positivity rate (Fig. 3I). The methylation status of O6-methylguanine DNA methyltransferase (*MGMT*) could not be examined because specimens obtained from needle biopsy were too small to extract sufficient amounts of DNA.

**Fig. 4:**
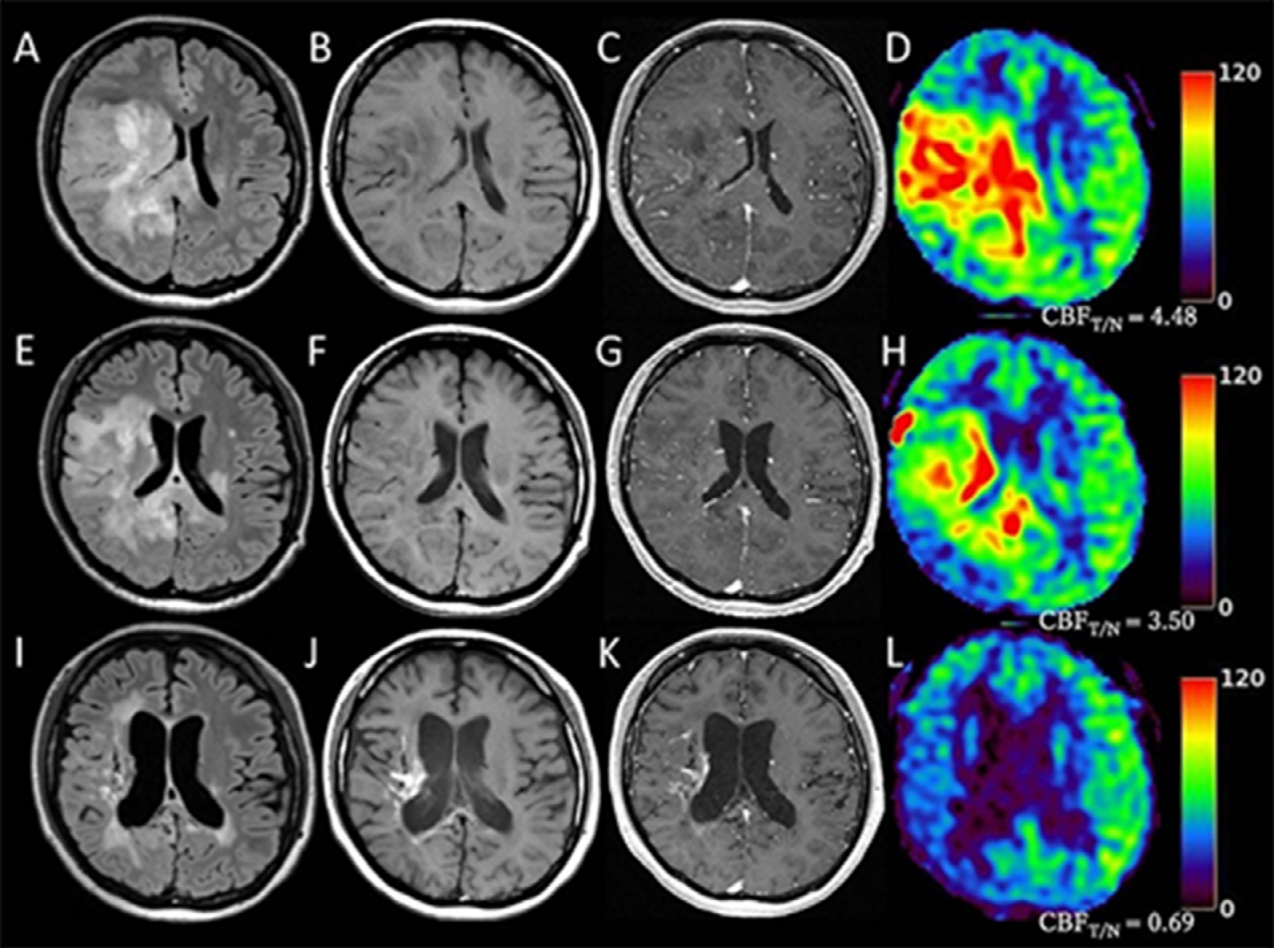
Imaging during treatment. (A-D) At the start of radiochemotherapy; (E-H) Two months after starting treatment; (I-L) 15 months after starting treatment. FLAIR (A, E, I), T1WI (B, F, J), Gd-T1WI (C, G, K), ASL (D, H, L).

During the 2 months between biopsy and diagnosis, the FLAIR-hyperintense area rapidly expanded, and high blood flow area enlarged on ASL with an increase in CBF_T/N_ (Figs. 4A-D). Together, hemiparesis of her left extremities deteriorated to the stage 0 of manual muscle test. Because we had concerns about permanent hemiparesis after reoperation for aggressive tumor resection as the tumor extended into areas of motor function, synchronized radiochemotherapy (extended local radiation at 60 Gy in 30 fractions and temozolomide at 75 mg/m^2^/day for the period of radiotherapy) was started at first. In addition, we administered bevacizumab (10 mg/kg, every 2 weeks) during the period of radiotherapy, because of the findings of high blood flow in the entire tumor on ASL, and the dense microvessels and VEGF expression in histological findings. Two months after starting combined radiochemotherapy, FLAIR showed significant reduction in tumor size, and ASL showed shrinkage of high blood flow areas with a significant decrease in CBF_T/N_ (Figs. 4E-H). Although her symptoms were unimproved, the patient was discharged from our hospital with a KPS of 50%. The patient was then treated with maintenance therapy using adjuvant temozolomide and bevacizumab at 15 mg/kg every 4 weeks in the outpatient department of our hospital. Although tumor shrinkage was maintained, residual tumor appearing hyperintense on FLAIR was identified in the white matter outside the lateral ventricle at 15 months after starting treatments (Fig. 4I). However, the finding of hypoperfusion in the entire residual tumor on ASL assured us of tumor remission. At the same time, strongly hyperintense areas suggesting growth of calcified lesions appeared on both T1WI and Gd-T1WI (Figs. 4I-K). According to findings from ASL, these calcifications were considered to represent reactive phenomena due to the therapy, rather than increased activity of the residual tumor. As of 20 months after starting initial treatment, the patient had continuously received maintenance therapy at KPS of 50% without tumor progression. Although enlargements of the cerebral ventricles were observed around 6 months after starting the initial treatment, neither intrathecal tumor dissemination nor idiopathic normal-pressure hydrocephalus was identified from head or spinal MRI or cytology of the cerebrospinal fluid. The ventricular enlargement was judged to reflect brain atrophy caused by therapies.

## Discussion

On conventional MRI, T2/FLAIR imaging shows nonspecific high signals in most patients with DHG, H3 G34m, while patients showing non-enhancement on Gd-T1WI are not uncommon. With regard to the frequency of non-enhancement or only faint enhancement such as in the present case, Puntonet et al. [7] reported 11 cases of DHG, H3 G34m, of whom 2 cases (18%) and 5 cases (45%) showed non-enhancement and faint enhancement, respectively, on Gd-T1WI. Furthermore, Picart et al. [4] reported either non- or faint enhancement in 11 of 15 cases (73%) of DHG, H3 G34m. Some reports have documented calcified foci on CT and MRI for DHG, H3 G34m, often in slow-growing tumors [6–8,10,13]. Kurokawa et al. [10] reviewed 59 cases of DHG, H3 G34m including 3 of their own cases, with 22.2% showing calcified foci on CT. Thus, conventional imaging shows non-specific findings but does detect calcified foci suggesting slow-growth tumors. That is why some patients with DHG, H3 G34m were initially suspected of non-neoplastic diseases such as demyelination, inflammation, or infection rather than malignancy [4,14]. Since supplementary neuroimaging modalities have long been desired, measures such as positron emission tomography [8] and magnetic resonance spectroscopy [4,15] for assessment of intratumoral metabolism, and dynamic susceptibility contrast perfusion MRI [10] and ASL [7,15] for assessment of intratumoral hemodynamics, have been applied to the diagnosis of DHG, H3 G34m. Whereas perfusion MRI is used with a contrast medium, ASL is a minimally invasive method with magnetically labeled arterial blood water as an endogenous tracer. ASL can evaluate intravascular blood flow without any influence from conditions of the blood-brain barrier (BBB) [16]. Although conventional MRI in this case showed only faint enhancement in part of the tumor, ASL demonstrated strong hyperperfusion throughout the entire tumor. This finding represented high vascular intensity despite preservation of the BBB, and led us to suspect highly malignant tumor.

The histological findings of DHG, H3 G34m have remained unclear, because this pathology shows a variety of histological features such as grade 3 astrocytoma [7,8], oligodendroglial appearance [4], anaplastic pleomorphic xanthoastrocytoma [5], and glial and dysplastic ganglion cell components [6], other than the main features of glioblastoma-like or embryonal tumor-like morphology. DHG, H3 G34m in this case mainly comprised low-differentiated tumor cells, and also contained abundant histiocytes and calcified foci. These findings resembled neither the glioblastoma-like nor embryonal tumor-like features frequently seen in DHG, H3 G34m. Histiocytes may be difficult to differentiate from oligodendroglioma cells. Since OLIG2 were not detected in the cells with clear cytoplasm and small bland nuclei, these cells were determined to represent histiocytes rather than oligodendroglioma cells. Further, the specimens in this case contained capillary networks with a chicken wire-like appearance. Although this finding was confirmed only by small specimens obtained from needle biopsy, we assumed that highly dense capillaries were likely present throughout the entire tumor, because ASL showed high blood flow in the entire tumor. In the literature, Puntonet et al. [7] and Onishi et al. [15] documented findings of ASL in 2 cases of DHG, H3 G34m in each report. The former reported high blood flow on ASL in both cases, whereas the latter reported low blood flow in both cases. Unsurprisingly, findings of ASL are likely to differ between each patient with DHG, H3 G34m, as those are necessarily influenced by the degree of intratumoral vascular structures. However, ASL can provide us with information for differential diagnosis of malignant tumors and also for an extension of intratumoral high flow vasculature, assuming that ASL shows hyperperfusion even in DHG, H3 G34m with preserved BBB as well as poor enhancement on Gd-T1WI, as seen in the present case. Routine ASL at the initial examinations should prove helpful in the diagnosis of DHG, H3 G34m.

The prognosis of DHG, H3 G34m is better than that of glioblastoma, with a median progression-free survival of 9 months and a median overall survival of 18-22 months [1,^3^,12]. The relatively favorable prognosis has been attributed to a high frequency (74%-79.5%) of *MGMT* methylation associated with the sensitivity to alkylating agents in patients with DHG, H3 G34m [3,17]. Although *MGMT* methylation could not be assessed for this case, the patient showed good therapeutic response and continued progression-free survival for 20 months after starting treatments including bevacizumab. Bevacizumab plays a role in restoration of the disrupted BBB in addition to inhibition of angiogenesis, thereby facilitating transport of other chemotherapeutic agents into the tumor [18]. Bevacizumab should have contributed to the success of treatment in this case. High blood flow on ASL has been reported to positively correlate with VEGF expression [19]. As shown in the present case, high blood flow on ASL predicts the presence of highly dense microvessels and expression of VEGF, even in DHG, H3 G34m showing non-contrast enhancement on Gd-T1WI, and probably allows physicians to choose bevacizumab for treatment.

Intratumoral calcifications in DHG, H3 G34m can be seen as high signals on T1WI [7]. In this case, the appearance of signal-hyperintense areas on T1WI in the chronic phase led us to have concerns about remnant activity in the tumor despite tumor shrinkage throughout treatment (Fig. 4J). However, 31.6% of patients with high-grade gliomas who received treatment with bevacizumab reportedly show intratumoral calcifications, because bevacizumab induces calcium accumulations in apoptotic cells [20,21]. From the point of view of hypoperfusion on ASL suggesting tumor remission, we speculated that enlarging calcifications in this case could represent reactive phenomenon due to bevacizumab, rather than being derived from tumor with remaining activities. ASL is thus useful also for keeping track of therapeutic responses with bevacizumab.

## Patient consent

Written informed consent was obtained from the patient for publication of this case report and accompanying images.
